# Healthcare consumers’ perceptions and experiences of an Australian prescription drug monitoring program: a cross-sectional survey

**DOI:** 10.3389/fdgth.2026.1745292

**Published:** 2026-09-17

**Authors:** Emma Tay, Meredith Makeham, Tracey-Lea Laba, Melissa Baysari

**Affiliations:** 1 Digital Health Human Factors Research Group, Faculty of Medicine and Health, The University of Sydney, Sydney, NSW, Australia; 2 Drug Health Services, Western Sydney Local Health District, Sydney, NSW, Australia; 3 Faculty of Medicine and Health, The University of Sydney, Sydney, NSW, Australia; 4 Faculty of Medicine, Health and Human Sciences, Macquarie University, Sydney, NSW, Australia; 5 Centre for Health Economics Research and Evaluation, The University of Technology Sydney, Sydney, NSW, Australia

**Keywords:** consumer, digital health, high risk medicines, implementation, medication safety, prescription drug monitoring program, prescription drugs

## Abstract

**Background:**

Prescription drug monitoring programs (PDMPs) are increasingly being recognised as a medication safety initiative to minimise harms from high-risk prescription medications. This study explored healthcare consumers’ experiences and perceptions of a state-wide PDMP in NSW, Australia, “SafeScript NSW”. We examined, 1) awareness and understanding of SafeScript NSW, and 2) perceptions of impacts of SafeScript NSW.

**Methods:**

Cross-sectional online survey was used; invitations were distributed via mailing lists and physical mailouts. Data collection occurred between May-August 2023, one year post roll-out of SafeScript NSW. All NSW-based consumers were eligible to participate. Rating scale and free-text response items were included. Descriptive statistics were used for analysis of quantitative data and a general inductive analysis approach used to code and identify themes from qualitative data.

**Results:**

77 consumers responses received. Two-thirds identified as female (51/77; 66.2%). Respondents were from all regions (metropolitan, regional, and rural) of NSW. Opioids and benzodiazepines were the top two PDMP-monitored medicines taken regularly. Most respondents (50/74; 67.6%) had not heard of SafeScript NSW prior to the survey and did not perceive it to be useful for identifying medicines-related harms (51/73; 69.9%). Close to half of the respondents noted no impacts of the system on their healthcare, including time spent at visits (10/22; 45.5%), or quality of care received (10/22; 45.5%). Over 50 free-text comments were received describing perceived impacts of SafeScript NSW which provided a more nuanced understanding of consumer experiences. The majority (59.6%) of these comments described negative experiences, including some reporting inability to access treatment and experiencing stigma.

**Conclusions:**

This study explored healthcare consumers’ experiences and perceptions of SafeScript NSW. Consumers had poor overall awareness and knowledge of SafeScript NSW and its impacts. Although primarily a practitioner-facing tool, our findings suggest additional dissemination of information and engagement of relevant consumers would foster consumer trust and confidence in their healthcare experiences and PDMP systems. Policies governing PDMP should account for the diverse needs of patients and practitioners. Realisation of PDMP's desired benefits should invite confidence and trust in care, and aim to avoid unintended harms to patients who may inadvertently be prevented from medically appropriate care.

## 1 Introduction

Harm management strategies related to high-risk prescription medicines are increasingly being supported by prescription drug monitoring programs (PDMPs). PDMPs, now implemented in the USA, Canada, some European countries, and Australia, vary in form, but generally are electronic systems that facilitate tracking or monitoring of prescription and/or dispensation data for certain medicines (1). Some PDMPs provide alerts to healthcare practitioners when certain scenarios or risk algorithms are met (1). Medicines monitored on PDMPs include those known to cause harms like overdoses and deaths (2–5). In New South Wales (NSW), Australia, a PDMP known as SafeScript NSW was rolled out across the state between October 2021 and May 2022. The medicines monitored on SafeScript NSW include opioids, benzodiazepines, stimulants, pregabalin and other controlled medicines (6). Use of SafeScript NSW by clinicians (prescribers and pharmacists) is currently non-mandatory, and clinicians can access the system for information to support safer clinical decision-making relating to use of monitored medicines (7). The SafeScript NSW system collects dispensing and prescribing data through the electronic prescription exchange services that connect to most dispensing and prescribing systems (2).

Our recent systematic review of reviews on PDMPs found that the majority of research evidence on PDMPs has primarily been generated from studies in the USA, and mostly focused on healthcare practitioners’ experiences (1). As PDMPs are increasingly used by healthcare practitioners to guide treatment discussions and decisions (8–11), these tools are likely to impact healthcare consumers (or patients), via changes to their healthcare management.

Research exploring healthcare consumers’ experiences of PDMP is scarce, and a similar gap has been observed locally in Australia. It has been suggested that more research is needed to understand consumer experiences of PDMPs (12). One previous small-scale Australian qualitative study undertaken in Victoria, another Australian state, reported that consumers expected PDMP would limit their access to extra-medical (non-prescribed indication) use of prescription medicines (13). They were also concerned about the consequences of limited access to medications, including withdrawal and inability to cope with psychosocial stressors (13). Additionally, the consumers reported experiencing stigma and viewed PDMP as negatively impacting therapeutic relationships (13).

In NSW, previous research has examined the experiences of the SafeScript NSW implementation team (14) and healthcare practitioners (15, 16), but no research to date has examined the perspectives of healthcare consumers. We examined in this study, 1) consumers’ awareness and understanding of SafeScript NSW, and 2) their perceptions of the impacts of SafeScript NSW.

## 2 Materials and methods

### 2.1 Study design

This study formed part of a larger mixed-methods PDMP evaluation to explore healthcare consumers’ and practitioners’ experiences and perceptions of SafeScript NSW. Here, we report on a cross-sectional online survey undertaken with healthcare consumers across NSW.

### 2.2 Survey instrument

The survey (Appendix A) was developed by a multi-disciplinary research team, comprising clinicians and researchers, including experts in human factors, digital health, pharmacy, general practice, health economics, clinical pharmacology, and addiction medicine. There were limited PDMP-related consumer surveys available in the literature, except for one survey undertaken in the Kentucky state of the USA that examined whether PDMP affected consumers’ access to controlled substance prescriptions (12). We were therefore required to develop a new survey instrument. Where possible, survey items were adopted/modified from those included in an Australian Bureau of Statistics consumer survey of healthcare in Australia (17), and the earlier consumer PDMP survey in the USA (12), and guided by SafeScript NSW online consumer resources (18). The survey was also informed by the nonadoption, abandonment, scale-up, spread, and sustainability (NASSS) framework (19) and the Quadruple Aim framework (20). We ensured relevant key domains of these frameworks were explored in the survey. The survey included 18 questions, including three with rating scales, and six with optional free-text responses to capture additional data. To discourage participants from abandoning the survey part way, all responses were optional. To assess face validity, the survey was reviewed by the research team and the SafeScript NSW program team prior to distribution to ensure it was usable and understandable. To ensure the survey was easy to navigate through, the instrument was pilot tested by a healthcare consumer. The survey included a participant information sheet (with study and data details), and participants were required to provide written consent prior to commencing the survey.

### 2.3 Study sample and recruitment

Recruitment of NSW-based healthcare consumers was undertaken via various channels. Healthcare consumer organisations (Appendix B) provided in-kind assistance with disseminating a survey advertisement or link to their members via their monthly newsletters and/or other member-based communication channels. The survey invitation was also disseminated by the SafeScript NSW program team to the consumer stakeholder organisations who assisted with SafeScript NSW's awareness and promotional efforts (Appendix B). Additionally, we utilised a paid mailout service by Services Australia, an Australian government social services agency, to distribute 2,500 survey invitations (including participant information sheet) to a randomly selected sample of healthcare consumers in NSW who were dispensed medicines that are on the SafeScript NSW monitored medicines list (Appendix C). The researchers did not have direct access to any of the distribution databases or lists, or to any data about potential participants. Participation was voluntary. No reimbursement was provided but participants were able to opt-in at the end of the survey to enter a lucky draw for one of two AUD20 gift vouchers.

### 2.4 Data collection

The survey was distributed one year following full SafeScript NSW state-wide roll out and was open for 3 months, between 29 May 2023 and 31 August 2023, date range was set *a priori*. The survey was administered online via the Qualtrics platform across 13 pages, with each page containing between one and three questions.

### 2.5 Data synthesis and analysis

Descriptive statistics were used for the analysis of quantitative data and a general inductive analysis approach was used to code and identify themes from free-text qualitative data. Free-text responses were reviewed by two researchers (ET and MB) who met to assign relevant codes to each comment via a discussion and consensus process. Codes were then grouped into themes and categorised as positive or negative perceptions.

This study was approved by the University of Sydney’s Human Research Ethics Committee.

## 3 Results

77 consumers responses were received. Two-thirds of the respondents identified as female (51/77; 66.2%). Not all participants responded to all questions although the average response rate for an individual item was 95.4% (range 87.5%–100%). We report results as proportions of those who answered each question. 68 unique postcodes were provided by 74 respondents. These postcodes encompassed all regions (metropolitan, regional, and rural) of NSW.

When asked what chronic conditions consumers experienced, six of 75 respondents reported not experiencing any of the chronic health conditions listed. Approximately half of the 69 respondents with chronic health conditions reported experiencing chronic mental health conditions (35/69; 50.7%) and musculoskeletal related conditions (32/69; 46.4%). Seventeen respondents provided additional free-text data on their other chronic health conditions, with lung disease (including asthma and chronic obstructive pulmonary disease) being most frequently listed (*n* = 4), followed by migraine and diabetes (both *n* = 3). Other than General Practitioners (GPs), other specialists, and pharmacies, a small number of respondents reported engaging with allied health clinicians such as physiotherapists (23/76; 30.3%) and psychologists (12/76; 15.8%). Table 1 outlines the demographics of respondents.

**Table 1:** Survey respondent demographics.

Demographics	n (%)
Age group (years)	77 (100)
18 to 24	7 (9.1)
25 to 34	8 (10.4)
35 to 44	4 (5.2)
45 to 54	17 (22.1)
55 to 64	16 (20.8)
65 to 74	16 (20.8)
75 to 84	8 (10.4)
85 or over	1 (1.3)
Gender	77 (100)
Female	51 (66.2)
Male	25 (32.5)
Prefer not to say	1 (1.3)
Non-binary/third gender	0 (0)
Other	0 (0)
Healthcare setting engagements^a^	76 (100)
General practitioner medical practice	72 (94.7)
Pharmacy	51 (67.1)
Private specialist clinic	35 (46.1)
Physiotherapist	23 (30.3)
Public hospital specialist clinic	18 (23.7)
Psychologist	12 (15.8)
Other	8 (10.5)
Community health centre	4 (5.3)
Chiropractor	4 (5.3)
Chronic health conditions^a^	75 (100)
Mental health condition (including anxiety disorder, depression, bipolar disorder, schizophrenia, post-traumatic stress disorder)	35 (46.7)
Arthritis (including osteoarthritis, rheumatoid arthritis, or lupus)	32 (42.7)
Other long-term health condition or injury	31 (41.3)
Chronic pain—moderate or severe pain (including nerve or neuropathic pain)	30 (40.0)
Insomnia/sleeping difficulties	24 (32.0)
Attention deficit hyperactivity disorder	15 (20.0)
Cancer (active or current)	4 (5.3)
Seizure disorder/epilepsy	1 (1.3)
Narcolepsy	0 (0)
None of the above	6 (8.0)

a multiple selections possible.

When reporting usual patterns of care sought from GPs, the majority of the survey respondents indicated they mostly saw one regular GP (56/76; 73.7%) (Table 2). Most of the 76 respondents (94.7%) reported they also see at least one other non-GP specialist (Table 3). Most (53/76; 69.7%) respondents regularly obtained their prescription medicines from one pharmacy (Table 4).

**Table 2:** Number of general practitioner (GP) specialists usually seen.

Number (*n* = 76)	n (%)
Mostly see one regular GP	56 (73.7)
Visit one medical practice and may see different GPs in the same practice	12 (15.8)
Visit different medical practices and see different GPs	6 (7.9)
Do not see any GP	2 (2.6)

**Table 3:** Number of other non-general practitioner (non-GP) specialists seen in a typical year.

Number (*n* = 76)	n (%)
None	4 (5.3)
1 to 2	45 (59.2)
3 to 4	21 (27.6)
5 or more	6 (7.9)

**Table 4:** Number of pharmacies for prescription medicines.

Number (*n* = 76)	n (%)
One regular pharmacy	53 (69.7)
Different pharmacies	21 (27.6)
Do not take any prescription medicines	2 (2.6)

Sixteen (21.9%) of 73 respondents reported not taking any of the listed medicines monitored by SafeScript NSW. Of the 57 respondents who did, opioids (26/57; 45.6%) and benzodiazepines (16/57; 28.1%) were the top two PDMP-monitored medicines reported to be taken on a regular and ongoing basis (Table 5). Eleven respondents provided additional information in the free-text section regarding other medicines used. Non-opioid analgesics (e.g., paracetamol and non-steroidal anti-inflammatory drugs) (*n* = 7), antidepressants (*n* = 5), and melatonin (*n* = 2) were frequently mentioned.

**Table 5:** Types of prescription medicines taken on a regular ongoing basis.

Medicines^a^ (*n* = 57)	n (%)
Opioids	26 (45.6)
Benzodiazepines	16 (28.1)
Other medicines including ketamine, pregabalin, quetiapine, cannabis-based medicines	15 (26.3)
Other medicines for pain/anxiety/sleep/ ADHD/narcolepsy not listed. Optional free-text	15 (26.3)
Medicines for ADHD or narcolepsy	12 (21.1)
Other sleeping aids	7 (12.3)

ADHD, attention deficit hyperactivity disorder.

a multiple selections possible.

### 3.1 Awareness and understanding of SafeScript NSW

Most (67.6%; *n* = 50) of the 74 respondents had not heard of SafeScript NSW prior to the survey. Of the 24 respondents who had heard of SafeScript NSW, the majority (17/24; 70.8%) reported they were taking monitored medicines listed in Table 5 while five (20.8%) reported not taking any monitored medicines listed and two did not disclose. 37.5% (9/24) of the 24 respondents reported hearing about SafeScript NSW from the medical practice or specialist clinic they attend (Appendix E Table 1). Seven respondents selected ‘other’, with four reporting they had heard of SafeScript NSW via television, news, or media. Overall, most of the survey respondents who had heard of SafeScript NSW rated their knowledge of the system between fair (7/23; 30.4%) and none (7/23; 30.4%) (Appendix E Table 2). 60.9% of the 23 respondents who had heard of SafeScript NSW were unsure whether SafeScript NSW is used by the medical practices or pharmacies they attend (Table 6).

**Table 6:** Knowledge of SafeScript NSW being used by medical practices or pharmacies attended.

Knowledge of use (*n* = 23)	n (%)
Yes	7 (30.4)
No	1 (4.4)
Not sure	14 (60.9)
Not applicable—I do not attend any	1 (4.4)

Of those who were aware of SafeScript NSW, the majority (13/22; 59.1%) of the respondents had not discussed findings from SafeScript NSW with their healthcare practitioners (Appendix E Table 3).

### 3.2 Perceived impacts of SafeScript NSW

We report perceived impacts, both from structured survey items and free-text responses, as 1) impacts on individual consumers and 2) impacts on the wider health system.

#### 3.2.1 Individual consumers

With respect to perceived changes to aspects of their healthcare management following SafeScript NSW implementation, more than half of the 22 respondents who were aware of SafeScript NSW noted that there had been no change in frequency of visits (14/22; 63.6%), or the frequency of referrals to other specialists (14/22; 63.6%) (Figure 1). Close to half also reported no change in quality of care received (10/22; 45.5%) or time spent at each visit (10/22; 45.5%) (Figure 1).

**Figure 1:**
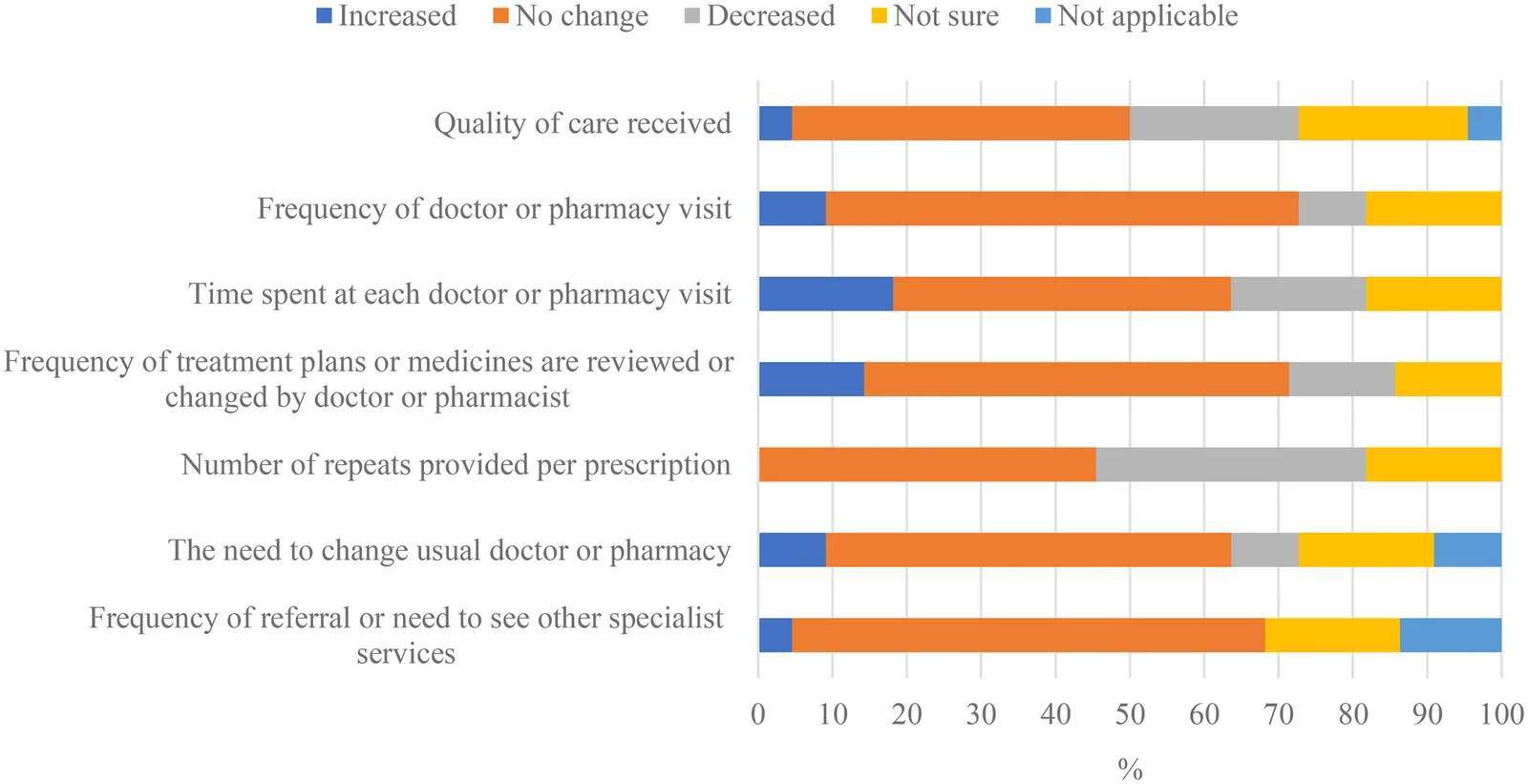
Perceived changes to own healthcare management following SafeScript NSW roll-out.

Ten respondents provided an additional 17 comments on the impacts of SafeScript NSW implementation on their healthcare. Via our thematic analysis approach, these were determined to be impacts on ‘people’ including emotions, behaviours, and knowledge, and impacts on ‘care’ including changes to individual care. Impacts on care were mentioned 9 times by 7 respondents, with most (*n* = 8) describing negative impacts. Table 7 provides further details with illustrative quotes.

**Table 7:** Perceived impacts of SafeScript NSW on individual consumers with illustrative quotes.

Themes	Examples
People (*n* = 8) Comments reflecting SafeScript NSW impacts on perceptions, attitudes, and understanding Positive comments (*n* = 4) Negative comments (*n* = 4)	Emotions (+): Less stress having to go into the doctors. (-): …and lastly it has meant I haven't sought medical assistance for issues from fear of stigma etc. Awareness or knowledge (+): Increased awareness of prescription monitoring. Behaviour (+): …now I ration medication and fill scripts so I do not run out and can go longer before seeking repeat prescriptions. (-): Not getting treatment at the local hospital as they can see I take pain meds and I am treated like a drug addict.
Care (*n* = 9) Comments related to SafeScript NSW impacts on individual care Positive comments (*n* = 1) Negative comments (*n* = 8)	(+): More affordable health care. (-): It has affected me very negatively at the beginning of the program. It made me go without my pain killers and withdraw which caused major problems physically and mentally. (-): It's a bad system where your specialist for a painful condition can't give you a script for meds that allow you to function and go to work safely because they are scared it’ll be flagged.

(+), positive comment; (-), negative comment.

#### 3.2.2 The wider health system

When asked about usefulness of SafeScript NSW in identifying harms related to monitored medicines, most (60.3%; 44/73) of the respondents indicated it was not useful. This included half or more of those with prior knowledge of SafeScript NSW (50%; 12/24), and those without (64%; 32/50). Approximately one-third (30.1%; 22/73) reported SafeScript NSW was useful in identifying harms related to monitored medicines (Figure 2).

**Figure 2:**
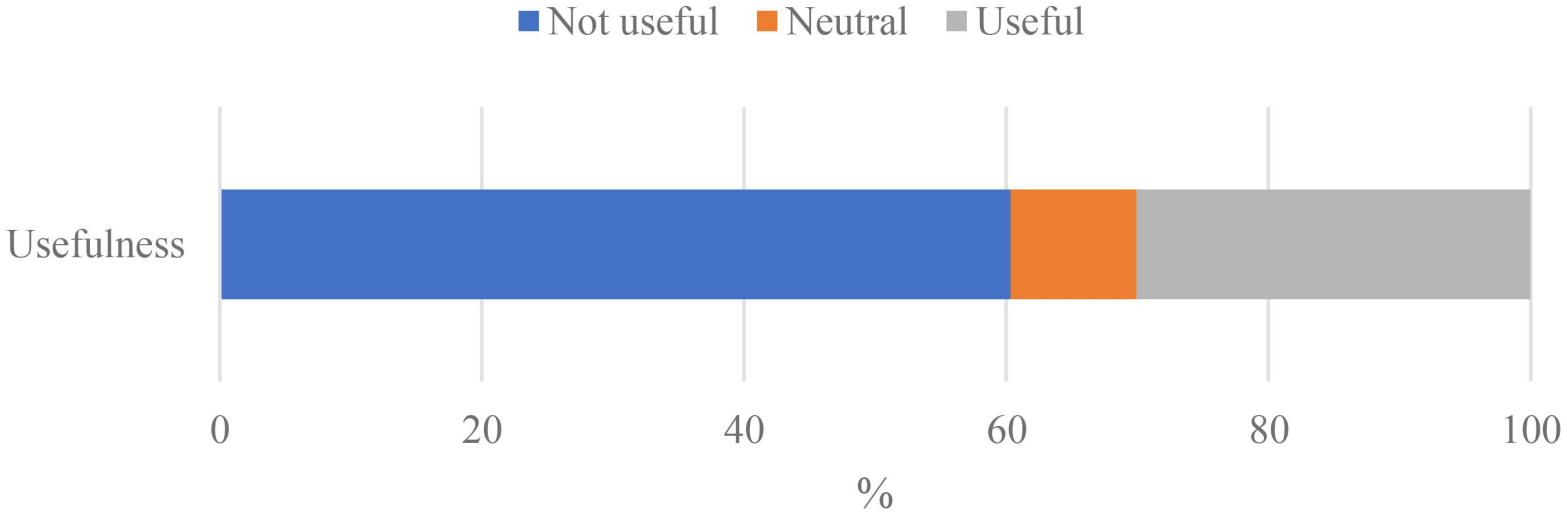
Perceived usefulness in identifying monitored medicines-related harms in the community.

### 3.3 Overall perceptions of SafeScript NSW

Participants were invited to provide additional free-text comments about SafeScript NSW at the end of the survey and 26 respondents provided this feedback. Some respondents were aware (11/26; 42.3%) and some were unaware (15/26; 57.7%) of SafeScript NSW prior to this study. A total of 40 comments were categorised into five broad themes—people, system, context, outcome, and implementation strategies. Each comment was classified as a positive or negative perception, with some respondents also making recommendations (Table 8). More than half (55%; 22/40) of the comments were negative.

**Table 8:** Overall perceptions of SafeScript NSW with illustrative quotes.

Themes	Examples
People (*n* = 23) Comments reflecting perceptions, attitudes, and understanding of SafeScript NSW Positive comments (*n* = 9) Negative comments (*n* = 14)	Perceptions (+): I think it is a good idea—it may help to find individuals that need further help, not just medication.—[N] Attitudes (+): This concept is so amazing to get quality health care without having to go into the doctors as often.—[A] Awareness or knowledge (+): Over the years have heard of patients going Doctor shopping and not for the right reasons to their conditions. I take my medications as directed by GP, Pain Management Specialist and Pharmacy so I believe SafeScript NSW will help in appropriate safeguarding of medications to those who need it.—[N] (-): It seems to me like this is targeted at people abusing certain medications. I don't believe there can be a really big case for the ’safety’ aspect—how many times do people really get the wrong combinations? Depending on how this is implemented it could lead to increased stigma around certain medications.—[N] (Rec): Will patients be advised if their medical practitioners etc will be using the SafeScript NSW system? I think it might be useful if patients are aware but also to understand what might be trigger and how there might be a way to clarify any matters. For example, if you stop seeing a doctor for other genuine reasons.—[N]
System (*n* = 4) Comments related to SafeScript NSW system features Positive comments (*n* = 2) Negative comments (*n* = 1) Recommendation (*n* = 1)	(+): It seems fairly safe from being mis-used.—[N] (-): I understand its usefulness, but it's a system that can be very easily abused.—[N] (Rec): There needs to be allowances for people who have to see different doctors in the same practice because the patient is a shift worker, Drs go on leave due to studying, sick leave or maternity leave. While it appears you may be Dr shopping, you are just trying to see “a” Dr in your practice.—[A]
Context (*n* = 5) Comments related to perceived relevance to own healthcare experience Positive comments (*n* = 2) Negative comments (*n* = 2) Recommendation (*n* = 1)	(+): It sounds like a good idea. Especially for those who have an addictive personality and do visit multiple pharmacies and doctors for opioids.—[N] (+): Living in Regional NSW, it is VERY difficult to access General Practitioners when required … The use of SafeScript (NSW) will assist by ensuring there is limited opportunity to misuse high risk prescription drugs, but also allow patients who have a need for these medications to ensure they are able to have a continued and stress free supply in the VERY common circumstance they are unable to access their normal GP.—[N] (-): It is absolutely devastating for many people experiencing complex health problems. It has caused professionals to be risk adverse and pharmacists unwarrant (sic) amount of power.—[A] (-): SafeScript (NSW) is used against people with chronic pain.—[A] (Rec): The individual person has to be at the forefront of this—blanket decisions that “this person is taking too much” cannot be made.—[N]
Implementation strategies (*n* = 2) Comments related to how SafeScript NSW was introduced Negative comments (*n* = 2)	(-): It obviously has not been well marketed.—[N] (-): It sounds good in theory but could cross privacy issues for the individual.—[N]
Outcomes (*n* = 6) Comments related to the impact of SafeScript NSW on patients, clinicians, and wider health system, including stigma Positive comments (*n* = 3) Negative comments (*n* = 3)	Wider system (+): It will help prevent accidental overdose and interaction which may put the safety of the patient and community at risk.—[A] Individual (+): I think it is an excellent initiative. Of little benefit for me other than reassurance as I am privileged to have been in at the beginning.—[A] (-): It is stigmatising and overall created issues for myself, my family and my broader community.—[A]

(+), positive comment; (-), negative comment; (Rec), recommendation; [A] respondents who had heard of SafeScript NSW prior to this study; [N] respondents who had not heard of SafeScript NSW prior to this study; GP, general practitioner; NSW, new south wales.

## 4 Discussion

Our survey of healthcare consumers revealed that awareness and understanding of SafeScript NSW is low, with most respondents reporting having no knowledge of the system prior to taking the survey. For those who knew of SafeScript NSW, most were unaware whether it was being used by the health services they attend and only four reported having had a discussion with their healthcare practitioners regarding SafeScript NSW or its findings. Most respondents also felt that SafeScript NSW had had a limited impact on their care and on the wider health system. This finding was comparable to a previous survey study in the USA where the majority (90%) of the consumer respondents reported being unaffected by PDMP (12).

Despite most of the results from the survey rating scales indicating limited impact of SafeScript NSW to care, the qualitative quotes revealed more nuances to respondents’ experiences and perceived impacts. The free-text feedback was largely negative and described negative impacts of PDMP on healthcare experiences. Some respondents alluded to restrictions or inability to access care and treatment sought, experiencing stigma, and avoiding care to prevent negative encounters. Negative consequences of PDMP have similarly been reported in other studies, despite differences in PDMP jurisdictions and access mandates. Barriers to treatment and/or medication access (12, 13, 21), increased stigma (13, 22), and strained therapeutic relationships (13, 22, 23) have been reported. Stigma can lead to impacts not only on the consumer in terms of poorer health outcomes or altered or reduced healthcare utilisation (24, 25), but also the wellbeing and performance of the healthcare workforce (24, 26, 27). It has been suggested that stigma towards people who use drugs or with substance use disorders should be carefully considered when implementing policies that promote or mandate the use of PDMPs (22). On the other hand, PDMP use may increase engagement between primary care practitioners and people at risk of or with substance use disorders (22), facilitate communication and collaboration between healthcare practitioners, and with patients, including some using PDMP as an opportunity to address potential medication use disorder or safety issues (9–11). Equipping practitioners with the preparedness and skills to navigate these encounters may improve quality of healthcare delivery (24) and minimise negative unintended consequences (e.g., stigma, abrupt or unexpected treatment cessation or refusal). Understanding drivers of stigma (including negative attitudes, knowledge and ability to manage conditions) and thence strategies to address these is crucial. Suggested strategies include information provision (e.g., presentations, lectures, pamphlets), skills-building activities (e.g., training workshops), participatory learning (e.g., discussions, role-play), and structural or policy changes (e.g., service integration, redress systems) (26). These can be targeted at healthcare practitioners and involve consumers where relevant (26).

Our study respondents reported greater awareness of prescription shopping and risks of medication-related harms following PDMP, with some implementing careful management of their medication supplies. Positive unintended consequences have also been reported in previous PDMP studies, such as greater awareness of substance use disorders, proactive monitoring or pharmacovigilance, and better understanding of population-level of controlled medicines use practices (21). Additionally, our survey of prescribers and pharmacists undertaken concurrently alongside this survey revealed that most practitioners perceived PDMP had improved quality of care, albeit with a corresponding increase in time spent on patient care encounters. This however contradicted our consumer respondents’ perceptions where most did not perceive changes to care, that is, no increase in frequency or length of time for visits, or quality of care received. This discrepancy may be because whilst added work is undertaken in the background by practitioners using SafeScript NSW, it may not necessarily result in direct discussions with consumers particularly if/when no concerns or alerts were triggered or if the practitioner did not see the need to discuss their use of PDMP. It is also likely that not all practitioners access SafeScript NSW data prior to prescribing or dispensing medications, as the system is non-mandatory. Thus, SafeScript NSW's impacts may not be visible or apparent to consumers. Furthermore, one study suggested that clinicians may avoid discussing PDMP data altogether following the discovery of a concerning history on PDMP (9), with some clinicians invoking alternative treatment strategies or policies (9, 28). Studies have also shown that some clinicians reportedly struggled with using PDMP data to inform decision-making or talk to patients, which has been attributed to a lack of training (9, 29).

It is interesting to note that consumers in our study did not view SafeScript NSW to be useful for identifying medicines related harms. This is in contrast to healthcare providers’ perceptions of PDMP in previous studies (8, 10, 14, 15, 23). Many of the free-text comments entered by respondents in our survey represented negative perceptions of SafeScript NSW, with only a small number (36.8%; 21/57 comments) reporting positive experiences and indicating they were supportive of its use. Previous research has highlighted that a balance that must be achieved between realising the goals/benefits of PDMPs in reducing or preventing medication related harms, and ensuring uncompromised access to medically appropriate treatment/care for consumers (12, 13). Practitioners should be encouraged to initiate conversations regarding PDMP information with consumers and provide an open, safe, and non-judgmental environment, inviting consumers to discuss relevant concerns (13). Furthermore, transparency and effective communication by healthcare providers has been shown to build patient trust and mitigate unintended effects such as stigma and strained therapeutic relationships (30). Open discussions between practitioners and consumers can invariably provide opportunities for information exchange and engagement of consumers to gain more knowledge and understanding regarding PDMP and its role in their healthcare management.

Another important result that emerged from our study was that the value of SafeScript NSW was reported to vary depending on context. For example, some respondents found that the availability of PDMP data helped with care continuity and treatment access when seeing different prescribers, particularly in regional areas, while others called for a more nuanced approach to triggering a ‘doctor shopping’ (multiple provider) alert in SafeScript NSW. The various healthcare delivery contexts and complexities involved in providing effective patient care should be carefully considered when developing PDMP related monitoring regulations and policies (23). The fact that PDMP was useful or relevant in some contexts but not others was raised in our study of healthcare practitioners (prescribers and pharmacists) where some perceived PDMP to be irrelevant in certain practice contexts e.g., prescribing in a residential aged care facility (15). Furthermore, the effectiveness of PDMPs in all populations has not yet been established (1). For example, there are a lack of studies demonstrating effectiveness of PDMP from the perspective of people who use drugs or with substance use disorders who experience pain (27). Most literature on opioid-related outcomes of PDMPs report mixed or inconsistent results (1, 21, 31–34), and PDMPs’ impact on rates of multiple provider is also variable (35). Collectively these findings indicate that PDMP is not a one-size-fits all solution, and it is likely to be more useful and effective in particular contexts, which has implications for mandating its widespread use.

### 4.1 Limitations

Our study was an opt-in online survey and we do not have access to the distribution lists or numbers reached by the consumer organisations who assisted with our survey distribution, preventing a response rate to be determined. We recruited only 77 participants, which was not based on when data saturation was achieved, and findings may not be representative of the views held by the diverse NSW healthcare consumer population. Approximately 90% of the respondents reported attending a single GP practice, in contrast to previous Australian research (36), which may suggest our sample is not representative of the general population. To some extent, we targeted participants who were taking high-risk monitored medicines, but these consumers represent a cohort with complex health conditions and care needs that may limit their ability to participate in the survey. Survey respondents were also self-selected and likely reflected those who had higher level of health literacy, were able and willing to participate in a web-based survey, and able to express their thoughts and interest in the implementation of SafeScript NSW. Those who had no prior knowledge of SafeScript NSW were potentially less likely to participate, although we found a general lack of awareness among respondents of our survey. SafeScript NSW use was optional for clinicians (prescribers and pharmacists) at the time of the survey, and the system may not have been utilised by participants’ treating clinicians. This potentially impacted participants’ awareness of this system as SafeScript NSW and its outputs were unlikely to be raised by clinician non-adopters. Survey items were non-compulsory to avoid early abandonment and to encourage participants to attempt as many questions as possible but as a result, not all participants answered all questions. However, the average response rate for individual items was reasonably high at 95.4%. Complementing rating scales with free text response items gave us greater insight into consumers’ experiences but we appreciate responses were optional and not all respondents completed these items, and respondents that did were limited by what they can enter into a survey box. Our future research will explore in-depth consumers’ perspectives of PDMP using qualitative methods.

## 5 Conclusions

This study explored healthcare consumers’ experiences and perceptions of a state-wide PDMP system one-year following roll-out. We found consumers had poor awareness and knowledge of SafeScript NSW, and most consumers did not perceive the tool as useful in identifying harms related to medicines monitored. Respondents who were aware of SafeScript NSW mostly reported that the tool had had no impact on their individual care. Qualitative free-text responses revealed a more nuanced and predominantly negative perception of PDMP from consumers. Despite being a practitioner-facing tool, its use can both directly and indirectly affect consumers’ care and outcomes, and subsequent health services utilisation and efficiency. This was seen in respondents who described both intentional and unintentional changes to their behaviour in their use of medications and health services. Our findings suggest additional information dissemination and engagement of relevant consumers would foster consumer trust and confidence in their healthcare experiences and PDMP systems. Further work is also needed to avoid and manage unintended consequences of PDMP implementation both from an individual and wider population perspective. Policies governing PDMP and strategies in place to support its use should take into account the diverse needs of both patients and practitioners, while recognising that PDMP may not be useful in all practice environments or contexts. Finally, our findings suggest that the realisation of PDMP's desired benefits should invite confidence and trust in care, and aim to avoid unintended harms to patients who may inadvertently be prevented from accessing required and medically appropriate care.

## Data Availability

The raw data for this study will not be shared, as we do not have permission from the participants or ethics approval to do so. Requests to access the datasets should be directed to etay5197@uni.sydney.edu.au.

## References

[1] Tay E Makeham M Laba T-L Baysari M. Prescription drug monitoring programs evaluation: a systematic review of reviews. Drug Alcohol Depend. (2023) 247:109887. 10.1016/j.drugalcdep.2023.109887 37126936

[2] NSW Department of Health. What is SafeScript NSW?. Available online at: https://www.health.nsw.gov.au/pharmaceutical/safescript/practitioners/Pages/what-is-safescript-nsw.aspx (Accessed May 4, 2025)

[3] Penington Institute. Overdose Snapshot 2026. Melbourne: Penington Institute (2026).

[4] Australian Bureau of Statistics. Deaths from external causes. (2024). Available online at: https://www.abs.gov.au/statistics/health/causes-death/deaths-external-causes/2024 (Accessed July 24, 2026)

[5] Evoy KE Sadrameli S Contreras J Covvey JR Peckham AM Morrison MD. Abuse and misuse of pregabalin and gabapentin: a systematic review update. Drugs. (2021) 81(1):125–56. 10.1007/s40265-020-01432-7 33215352

[6] NSW Department of Health. SafeScript NSW—list of monitored medicines. (2024). Available online at: https://www.health.nsw.gov.au/pharmaceutical/safescript/Pages/monitored-medicines.aspx(Accessed July 29, 2024)

[7] NSW Department of Health. How and when to use SafeScript NSW. (2024). Available online at: https://www.health.nsw.gov.au/pharmaceutical/safescript/practitioners/Pages/How-to-use-safescript.aspx (Accessed July 20, 2026).

[8] Picco L Lam T Haines S Nielsen S. How prescription drug monitoring programs influence clinical decision-making: a mixed methods systematic review and meta-analysis. Drug Alcohol Depend. (2021) 228:109090. 10.1016/j.drugalcdep.2021.109090 34600255

[9] Hildebran C Cohen DJ Irvine JM Foley C O'Kane N Beran T. How clinicians use prescription drug monitoring programs: a qualitative inquiry. Pain Med. (2014) 15(7):1179–86. 10.1111/pme.12469 24833113 PMC4180422

[10] Hoppe D George Liu C Khalil H. Health-care practitioner use of prescription drug monitoring programs in clinical practice in Australia: a qualitative study. Drug Alcohol Rev. (2023) 42(7):1647–57. 10.1111/dar.13711 37402606

[11] Rutkow L Smith KC Lai AY Vernick JS Davis CS Alexander GC. Prescription drug monitoring program design and function: a qualitative analysis. Drug Alcohol Depend. (2017) 180:395–400. 10.1016/j.drugalcdep.2017.08.040 28978492

[12] Goodin A Blumenschein K Freeman PR Talbert J. Consumer/patient encounters with prescription drug monitoring programs: evidence from a medicaid population. Pain Physician. (2012) 15(3 Suppl):Es169–75. 10.36076/ppj.2012/15/ES169 22786455 PMC3545447

[13] Haines S Lam A Savic M Carter A. Patient experiences of prescription drug monitoring programs: a qualitative analysis from an Australian pharmaceutical helpline. Int J Drug Policy. (2022) 109:103847. 10.1016/j.drugpo.2022.103847 36067724

[14] Tay E Makeham M Hargreaves A Laba TL Baysari M. Prescription drug monitoring program in Australia: a qualitative study of stakeholders’ experiences and perceptions of a state-wide implementation. BMC Health Serv Res. (2024) 24(1):1147. 10.1186/s12913-024-11614-8 39343889 PMC11439230

[15] Tay E Makeham M Laba TL Raubenheimer J Baysari M. Healthcare practitioners’ perceptions and experiences of an Australian prescription drug monitoring program: a cross-sectional survey. Under Review. (2026).

[16] Tay E Makeham M Baysari M. Implementation of a statewide prescription drug monitoring program—healthcare Practitioners’ perspectives. Stud Health Technol Inform. (2026) 339:64–9. 10.3233/SHTI260966 42460594

[17] Australian Bureau of Statistics. Survey of health care 2016. (2016). Available online at: https://www.abs.gov.au/AUSSTATS/subscriber.nsf/log?openagent&4343.0%20survey%20of%20health%20care%202016%20questionnaire.pdf&4343.0&Publication&00C343D9D3867E1FCA2581A000206C9A&&2016&20.09.2017&Latest (Accessed August 17, 2021)

[18] NSW Department of Health. Safe script NSW for consumers (2024). Available online at: https://www.health.nsw.gov.au/pharmaceutical/safescript/consumers/Pages/default.aspx (Accessed November 18, 2024).

[19] Greenhalgh T Wherton J Papoutsi C Lynch J Hughes G A'Court C. Beyond adoption: a new framework for theorizing and evaluating nonadoption, abandonment, and challenges to the scale-up, spread, and sustainability of health and care technologies. J Med Internet Res. (2017) 19(11):e367. 10.2196/jmir.8775 29092808 PMC5688245

[20] Bodenheimer T Sinsky C. From triple to quadruple aim: care of the patient requires care of the provider. Ann Fam Med. (2014) 12(6):573–6. 10.1370/afm.1713 25384822 PMC4226781

[21] Yuanhong Lai A Smith KC Vernick JS Davis CS Caleb Alexander G Rutkow L. Perceived unintended consequences of prescription drug monitoring programs. Subst Use Misuse. (2019) 54(2):345–9. 10.1080/10826084.2018.1491052 30463465

[22] Allen B Harocopos A Chernick R. Substance use stigma, primary care, and the New York state prescription drug monitoring program. Behav Med. (2020) 46(1):52–62. 10.1080/08964289.2018.1555129 30726167

[23] Carnes NA Wright ER Norwood CW. A qualitative analysis of prescribers’ and dispensers’ views on improving prescription drug monitoring programs. Res Social Adm Pharm. (2017) 13(6):1167–74. 10.1016/j.sapharm.2016.12.002 28007411

[24] Van Boekel LC Brouwers EP Van Weeghel J Garretsen HF. Stigma among health professionals towards patients with substance use disorders and its consequences for healthcare delivery: systematic review. Drug Alcohol Depend. (2013) 131(1-2):23–35. 10.1016/j.drugalcdep.2013.02.018 23490450

[25] Muncan B Walters SM Ezell J Ompad DC. “They look at US like junkies": influences of drug use stigma on the healthcare engagement of people who inject drugs in New York city. Harm Reduct J. (2020) 17(1):53. 10.1186/s12954-020-00399-8 32736624 PMC7393740

[26] Nyblade L Stockton MA Giger K Bond V Ekstrand ML Lean RM. Stigma in health facilities: why it matters and how we can change it. BMC Med. (2019) 17(1):25. 10.1186/s12916-019-1256-2 30764806 PMC6376713

[27] Voon P Greer AM Amlani A Newman C Burmeister C Buxton JA. Pain as a risk factor for substance use: a qualitative study of people who use drugs in British Columbia, Canada. Harm Reduct J. (2018) 15(1):35. 10.1186/s12954-018-0241-y 29976203 PMC6034304

[28] Hildebran C Leichtling G Irvine JM Cohen DJ Hallvik SE Deyo RA. Clinical styles and practice policies: influence on communication with patients regarding worrisome prescription drug monitoring program data. Pain Med. (2016) 17(11):2061–6. 10.1093/pm/pnw019 27009292 PMC5035181

[29] Hong M Seymour S Stopka TJ Bandanza L Crocker E Morgan A. “Nobody knows how you're supposed to interpret it:” End-user perspectives on prescription drug monitoring program in Massachusetts. J Addict Med. (2022) 16(3):e171–e6. 10.1097/ADM.0000000000000901 34417413 PMC8857300

[30] Jones RD Krenz C Griffith KA Spence R Bradbury AR De Vries R. Patient experiences, trust, and preferences for health data sharing. JCO Oncol Pract. (2022) 18(3):e339–e50. 10.1200/OP.21.00491 34855514 PMC8932496

[31] Puac-Polanco V Chihuri S Fink DS Cerdá M Keyes KM Li G. Prescription drug monitoring programs and prescription opioid-related outcomes in the United States. Epidemiol Rev. (2020) 42(1):134–53. 10.1093/epirev/mxaa002 32242239 PMC7947593

[32] Ponnapalli A Grando A Murcko A Wertheim P. Systematic literature review of prescription drug monitoring programs. AMIA Annu Symp Proc. (2018) 2018:1478–87.PMCID: PMC6371270 30815193 PMC6371270

[33] Nielsen S Picco L Russell G Pearce C Andrew NE Lubman DI. Changes in opioid and other analgesic prescribing following voluntary and mandatory prescription drug monitoring program implementation: a time series analysis of early outcomes. Int J Drug Policy. (2023) 117:104053. 10.1016/j.drugpo.2023.104053 37209441

[34] Fink DS Schleimer JP Sarvet A Grover KK Delcher C Castillo-Carniglia A. Association between prescription drug monitoring programs and nonfatal and fatal drug overdoses: a systematic review. Ann Intern Med. (2018) 168(11):783–90. 10.7326/M17-3074 29801093 PMC6015770

[35] Wilson MN Hayden JA Rhodes E Robinson A Asbridge M. Effectiveness of prescription monitoring programs in reducing opioid prescribing, dispensing, and use outcomes: a systematic review. J Pain. (2019) 20(12):1383–93. 10.1016/j.jpain.2019.04.007 31059823

[36] Wright M Hall J Van Gool K Haas M. How common is multiple general practice attendance in Australia? Aust J Gen Pract. (2018) 47(5):289–96. 10.31128/AJGP-11-17-4413 29779298

