# Supplementary material for: Healthcare consumers’ perceptions and experiences of an Australian prescription drug monitoring program: a cross-sectional survey
Source: Front Digit Health. 2026 Sep 17;8:1745292. doi: 10.3389/fdgth.2026.1745292 (PMC13627383; doi:10.3389/fdgth.2026.1745292)
Supplement: Supplementary file 1 [file Datasheet1.docx]

**Supplementary File**

**Appendix A. Survey instrument**

| 1) What is your current age (years)? | **☐** 18 to 24  **☐** 25 to 34  **☐** 35 to 44  **☐** 45 to 54  **☐** 55 to 64  **☐** 65 to 74  **☐** 75 to 84  **☐** 85 or over |
| --- | --- |
| 2) What is your gender? | **☐** Female  **☐** Male  **☐** Non-binary/third gender  **☐** Other  **☐** Prefer not to say |
| 3) Where is your primary residential location in NSW? | **☐** Please enter postcode: **_________________** |
| 4) Which practice setting(s) do you attend for your health care? (Select all that apply) | **☐** Chiropractor  **☐** Community health centre  **☐** General Practitioner (GP) medical practice(s)  **☐** Pharmacy  **☐** Physiotherapist  **☐** Private specialist clinic(s)  **☐** Psychologist  **☐** Public hospital specialist clinic(s)  **☐** Other: **_________________________** |
| 5) How do you usually seek care from General Practitioners (GPs)? | **☐** I mostly see one regular GP  **☐** I visit one medical practice and may see different GPs in the same practice  **☐** I visit different medical practices and see different GPs  **☐** I do not see any GP |
| 6) How many different Medical Specialists (excluding GPs) do you see in a typical year for your health care? | **☐** None  **☐** 1 to 2  **☐** 3 to 4  **☐** 5 or more |
| 7) Which of the following best describes how you get prescription medicines? | **☐** I go to one regular pharmacy  **☐** I go to different pharmacies  **☐** I do not take any prescription medicines ***[skip to Q9]*** |
| 8) How many different medications from the list below are you taking on a regular and ongoing basis? (Select all that apply) Opioids pain relievers \| - Buprenorphine (e.g. Norspan/ Bupredermal patches, Temgesic, Suboxone) - Codeine (e.g. Panadeine, Panadeine Forte, Codeine, Mersyndol) - Fentanyl (e.g. Durogesic/ Dutran/ Fenpatch, Actiq lozenge, Abstral tablets) - Hydromorphone (e.g. Dilaudid, Jurnista) - Methadone (e.g. Physeptone, Biodone Forte) - Morphine (e.g. Kapanol, MS Contin, MS Mono, Ordine, Sevredol) - Oxycodone (e.g. Endone, OxyContin, OxyNorm, Targin) - Pethidine - Tapentadol (e.g. Palexia) - Tramadol (e.g. Tramal); Benzodiazepines (often prescribed for anxiety or sleep) \| - Alprazolam (e.g. Alprax, Kalma, Xanax) - Bromazepam (e.g. Lexotan) - Clobazam (e.g. Frisium) - Clonazepam (e.g. Rivotril, Paxam) - Diazepam (e.g. Valium, Antenex) - Flunitrazepam (e.g. Hypnodorm) - Lorazepam (e.g. Ativan) - Nitrazepam (e.g. Mogadon, Alodorm) - Oxazepam (e.g. Serepax, Murelax, Alepam) - Temazepam (e.g. Normison, Temaze); Other sleeping aids \| - Zolpidem (e.g. Stilnox) - Zopiclone (e.g. Imovane, Imrest, Imoclone); Medicines for attention deficit hyperactivity disorder (ADHD) or narcolepsy \| - Dexamfetamine - Lisdexamfetamine (e.g. Vyvanse) - Methylphenidate (e.g. Ritalin, Concerta, Artige); Additional medicines \| - Ketamine - Pregabalin (e.g. Lyrica) - Quetiapine (e.g. Seroquel, Quetia) - Cannabis-based medicines | ☐ Opioid pain relievers  ☐ Benzodiazepines (often prescribed for anxiety or sleep)  ☐ Other sleeping aids  ☐ Medicines for attention deficit hyperactivity disorder (ADHD) or narcolepsy  ☐ Additional medicines  ☐ I take other medicines for pain/anxiety/sleep/ADHD/narcolepsy that are not listed above (please specify if able) –  ☐ Not applicable - I do not take any of these medicines |
| 9) Do you have the following long-term or chronic health conditions? Chronic conditions are conditions that are expected to last or have already lasted 6 months or more and have been diagnosed by a health professional. (Select all that apply) | **☐** Arthritis (including osteoarthritis, rheumatoid arthritis, or lupus)  **☐** Attention deficit hyperactivity disorder (ADHD)  **☐** Cancer (active or current)  **☐** Chronic pain - moderate or severe pain (including nerve or neuropathic pain)  **☐** Insomnia/sleeping difficulties  **☐** Mental health condition (including anxiety disorder, depression, bipolar disorder, schizophrenia, post-traumatic stress disorder)  **☐** Narcolepsy  **☐** Seizure disorder/epilepsy  **☐** Other long-term health-condition or injury – (free text)  **☐** None of the above |
| SafeScript NSW is a computer software system that allows prescribers (doctors, nurse practitioners and dentists) and pharmacists to access real-time information about a patient’s prescription history for certain high-risk medicines such as opioids and benzodiazepines to help them make safer decisions about patient care and to keep patients safe when taking these medicines.  The medicines monitored in SafeScript NSW include the following: opioid pain relievers, medicines for anxiety or sleeping tablets (benzodiazepines), other sleeping tablets, psychostimulants for attention deficit hyperactivity disorder (ADHD) or narcolepsy, or other high-risk medicines.  You can access more information about SafeScript NSW here: <https://www.safescript.health.nsw.gov.au/> | |
| 10) Prior to taking this survey, had you heard of the SafeScript NSW system? | **☐** Yes  **☐** No ***[skip to Q15]*** |
| 11) From which source(s) did you hear about SafeScript NSW? (Select all that apply) | **☐** Medical practice/specialist clinic I attend  **☐** Internet  **☐** Newsletter from consumer bodies/groups  **☐** Word of mouth  **☐** Other (Please describe) **_________________** |
| 12) How would you rate your knowledge of SafeScript NSW? | **☐** 1 None  **☐** 2 Fair  **☐** 3 Good  **☐** 4 Very Good  **☐** 5 Excellent |
| 13) Do the medical practice(s) and/or pharmacy/ies you attend use the SafeScript NSW system? | **☐** Yes  **☐** No  **☐** Not sure  **☐** Not applicable – I do not attend any |
| 14) Had any of your doctors or pharmacists discussed a report or findings generated from the SafeScript NSW system with you? | **☐** Yes  **☐** No  **☐** Not sure  **☐** Not applicable |
| SafeScript NSW helps prescribers and pharmacists to identify circumstances where a patient may be at risk of harm. These circumstances include where a patient:   - has visited multiple prescribers and pharmacies within a short period of time - is taking high doses of opioid pain relievers - is taking two or more medicines that have potential to become harmful when combined, for example certain opioids (e.g. OxyContin^®^, Endone^®^ or fentanyl) and benzodiazepines (e.g. Valium^®^ or temazepam) | |
| 15) On a scale of 1 to 10, how useful do you think SafeScript NSW would be in identifying harms related to SafeScript NSW-monitored medicines in the community? | 1 (not at all useful) – 10 (very useful) 1 \| 2 \| 3 \| 4 \| 5 \| 6 \| 7 \| 8 \| 9 \| 10; ☐ \| ☐ \| ☐ \| ☐ \| ☐ \| ☐ \| ☐ \| ☐ \| ☐ \| ☐ |
| 16) [Those who answered “Yes” in Q10]  Based on your experience, how do you think SafeScript NSW has changed the following in your health care management?   - Quality of care received - How frequently you visit your doctor or pharmacy - Time spent at each doctor or pharmacy visit - How frequently your treatment plans or medicines are reviewed or changed by the doctor or pharmacist - The number of repeats provided per script - The need to change your usual doctor or pharmacy - How often you are referred to, or need to see other specialist services (e.g., chronic pain, mental health, or addiction medicine specialists) | Increased \| No change \| Decreased \| Not sure \| N/A; ☐ \| ☐ \| ☐ \| ☐ \| ☐; ☐ \| ☐ \| ☐ \| ☐ \| ☐; ☐ \| ☐ \| ☐ \| ☐ \| ☐; ☐ \| ☐ \| ☐ \| ☐ \| ☐; ☐ \| ☐ \| ☐ \| ☐ \| ☐; ☐ \| ☐ \| ☐ \| ☐ \| ☐; ☐ \| ☐ \| ☐ \| ☐ \| ☐ |
| 17) Can you think of any other impact SafeScript NSW has had on your health care? | (free text)  **_________________________________________**  **_________________________________________** |
| 18) Do you have any further comments you would like to make about the SafeScript NSW system? | (free text)  **_________________________________________**  **_________________________________________** |

**Appendix B. Healthcare consumer organisations that assisted in distributing survey advertisement (listed in alphabetical order)**

- Health Consumers NSW
- NSW Health – eHealth SafeScript NSW program team consumer stakeholders
- NSW Users and AIDS Association (NUAA)
- PainAustralia

**Appendix C. Services Australia mailout selection criteria**

Inclusion criteria:

Random sampling of consumers who reside in New South Wales and are prescribed under the Pharmaceutical Benefits Scheme (PBS) and Repatriation Pharmaceutical Benefits Scheme (RPBS) of the below target medicines between time period of 1 June 2022 to 30 November 2022.

This method of recruitment is limited by only things included in PBS - included general schedule & repatriation pharmaceutical benefits scheme (only one cannabidiol – Epidyolex)

Exclusion criteria:

Target medicines not on PBS: Clobazam, pethidine, lorazepam, zolpidem, ketamine, medicines as part of a prescriber bag, target medicines prescribed under “private” script (not subsidised or under PBS)

| Opioids | - Buprenorphine (e.g. Norspan/ Bupredermal patches, Temgesic, Suboxone)  - Codeine (e.g. Panadeine, Panadeine Forte, Codeine, Mersyndol)  - Fentanyl (e.g. Durogesic/ Dutran/ Fenpatch, Actiq lozenge, Abstral tablets)  - Hydromorphone (e.g. Dilaudid, Jurnista)  - Methadone (e.g. Physeptone, Biodone Forte)  - Morphine (e.g. Kapanol, MS Contin, MS Mono, Ordine, Sevredol)  - Oxycodone (e.g. Endone, OxyContin, OxyNorm, Targin)  - Pethidine  - Tapentadol (e.g. Palexia)  - Tramadol (e.g. Tramal) |
| --- | --- |
| Benzodiazepines | - Alprazolam (e.g. Alprax, Kalma, Xanax)  - Bromazepam (e.g. Lexotan)  - Clobazam (e.g. Frisium)  - Clonazepam (e.g. Rivotril, Paxam)  - Diazepam (e.g. Valium, Antenex)  - Flunitrazepam (e.g. Hypnodorm)  - Lorazepam (e.g. Ativan)  - Nitrazepam (e.g. Mogadon, Alodorm)  - Oxazepam (e.g. Serepax, Murelax, Alepam)  - Temazepam (e.g. Normison, Temaze) |
| Other sleeping aids | - Zolpidem (e.g. Stilnox)  - Zopiclone (e.g. Imovane, Imrest, Imoclone) |
| Medicines for attention deficit hyperactivity disorder (ADHD) or narcolepsy | - Dexamfetamine  - Lisdexamfetamine (e.g. Vyvanse)  - Methylphenidate (e.g. Ritalin, Concerta, Artige) |
| Additional medicines | - Ketamine  - Pregabalin (e.g. Lyrica)  - Quetiapine (e.g. Seroquel, Quetia)  - Cannabidiol (Epidyolex) |

**Appendix D. Checklist for Reporting Results of Internet E-Surveys (CHERRIES)**

| ***Checklist Item*** | ***Explanation*** | ***Page Number in paper*** |
| --- | --- | --- |
| Describe survey design | Describe target population, sample frame. Is the sample a convenience sample? (In “open” surveys this is most likely.) | 5-6 |
| IRB approval | Mention whether the study has been approved by an IRB. | 7 |
| Informed consent | Describe the informed consent process. Where were the participants told the length of time of the survey, which data were stored and where and for how long, who the investigator was, and the purpose of the study? | 6 |
| Data protection | If any personal information was collected or stored, describe what mechanisms were used to protect unauthorized access. | N/A |
| Development and testing | State how the survey was developed, including whether the usability and technical functionality of the electronic questionnaire had been tested before fielding the questionnaire. | 5-6 |
| Open survey versus closed survey | An “open survey” is a survey open for each visitor of a site, while a closed survey is only open to a sample which the investigator knows (password-protected survey). | 6 |
| Contact mode | Indicate whether or not the initial contact with the potential participants was made on the Internet. (Investigators may also send out questionnaires by mail and allow for Web-based data entry.) | 6 |
| Advertising the survey | How/where was the survey announced or advertised? Some examples are offline media (newspapers), or online (mailing lists – If yes, which ones?) or banner ads (Where were these banner ads posted and what did they look like?). It is important to know the wording of the announcement as it will heavily influence who chooses to participate. Ideally the survey announcement should be published as an appendix. | 6 |
| Web/E-mail | State the type of e-survey (eg, one posted on a Web site, or one sent out through e-mail). If it is an e-mail survey, were the responses entered manually into a database, or was there an automatic method for capturing responses? | 6 |
| Context | Describe the Web site (for mailing list/newsgroup) in which the survey was posted. What is the Web site about, who is visiting it, what are visitors normally looking for? Discuss to what degree the content of the Web site could pre-select the sample or influence the results. For example, a survey about vaccination on a anti-immunization Web site will have different results from a Web survey conducted on a government Web site | N/A |
| Mandatory/voluntary | Was it a mandatory survey to be filled in by every visitor who wanted to enter the Web site, or was it a voluntary survey? | 6-7 |
| Incentives | Were any incentives offered (eg, monetary, prizes, or non-monetary incentives such as an offer to provide the survey results)? | 7 |
| Time/Date | In what timeframe were the data collected? | 7 |
| Randomization of items or questionnaires | To prevent biases items can be randomized or alternated. | N/A |
| Adaptive questioning | Use adaptive questioning (certain items, or only conditionally displayed based on responses to other items) to reduce number and complexity of the questions. | Appendix A. Survey instrument |
| Number of Items | What was the number of questionnaire items per page? The number of items is an important factor for the completion rate. | 7 |
| Number of screens (pages) | Over how many pages was the questionnaire distributed? The number of items is an important factor for the completion rate. | 7 |
| Completeness check | It is technically possible to do consistency or completeness checks before the questionnaire is submitted. Was this done, and if “yes”, how (usually JAVAScript)? An alternative is to check for completeness after the questionnaire has been submitted (and highlight mandatory items). If this has been done, it should be reported. All items should provide a non-response option such as “not applicable” or “rather not say”, and selection of one response option should be enforced. | N/A |
| Review step | State whether respondents were able to review and change their answers (eg, through a Back button or a Review step which displays a summary of the responses and asks the respondents if they are correct). | N/A |
| Unique site visitor | If you provide view rates or participation rates, you need to define how you determined a unique visitor. There are different techniques available, based on IP addresses or cookies or both. | N/A |
| View rate (Ratio of unique survey visitors/unique site visitors) | Requires counting unique visitors to the first page of the survey, divided by the number of unique site visitors (not page views!). It is not unusual to have view rates of less than 0.1 % if the survey is voluntary. | N/A |
| Participation rate (Ratio of unique visitors who agreed to participate/unique first survey page visitors) | Count the unique number of people who filled in the first survey page (or agreed to participate, for example by checking a checkbox), divided by visitors who visit the first page of the survey (or the informed consents page, if present). This can also be called “recruitment” rate. | N/A |
| Completion rate (Ratio of users who finished the survey/users who agreed to participate) | The number of people submitting the last questionnaire page, divided by the number of people who agreed to participate (or submitted the first survey page). This is only relevant if there is a separate “informed consent” page or if the survey goes over several pages. This is a measure for attrition. Note that “completion” can involve leaving questionnaire items blank. This is not a measure for how completely questionnaires were filled in. (If you need a measure for this, use the word “completeness rate”.) | 7 |
| Cookies used | Indicate whether cookies were used to assign a unique user identifier to each client computer. If so, mention the page on which the cookie was set and read, and how long the cookie was valid. Were duplicate entries avoided by preventing users access to the survey twice; or were duplicate database entries having the same user ID eliminated before analysis? In the latter case, which entries were kept for analysis (eg, the first entry or the most recent)? | N/A |
| IP check | Indicate whether the IP address of the client computer was used to identify potential duplicate entries from the same user. If so, mention the period of time for which no two entries from the same IP address were allowed (eg, 24 hours). Were duplicate entries avoided by preventing users with the same IP address access to the survey twice; or were duplicate database entries having the same IP address within a given period of time eliminated before analysis? If the latter, which entries were kept for analysis (eg, the first entry or the most recent)? | N/A |
| Log file analysis | Indicate whether other techniques to analyze the log file for identification of multiple entries were used. If so, please describe. | N/A – Each survey response is allocated individual Response ID. |
| Registration | In “closed” (non-open) surveys, users need to login first and it is easier to prevent duplicate entries from the same user. Describe how this was done. For example, was the survey never displayed a second time once the user had filled it in, or was the username stored together with the survey results and later eliminated? If the latter, which entries were kept for analysis (eg, the first entry or the most recent)? | N/A |
| Handling of incomplete questionnaires | Were only completed questionnaires analyzed? Were questionnaires which terminated early (where, for example, users did not go through all questionnaire pages) also analyzed? | 7 |
| Questionnaires submitted with an atypical timestamp | Some investigators may measure the time people needed to fill in a questionnaire and exclude questionnaires that were submitted too soon. Specify the timeframe that was used as a cut-off point, and describe how this point was determined. | N/A |
| Statistical correction | Indicate whether any methods such as weighting of items or propensity scores have been used to adjust for the non-representative sample; if so, please describe the methods. | N/A |

This checklist has been modified from Eysenbach G. Improving the quality of Web surveys: the Checklist for Reporting Results of Internet E-Surveys (CHERRIES). J Med Internet Res. 2004 Sep 29;6(3):e34 [erratum in J Med Internet Res. 2012; 14(1): e8.]. Article available at [https://www.jmir.org/2004/3/e34](https://www.jmir.org/2004/3/e34/)/; erratum available <https://www.jmir.org/2012/1/e8/>. Copyright ©Gunther Eysenbach. Originally published in the [Journal of Medical Internet](http://www.jmir.org) Research, 29.9.2004 and 04.01.2012.

**Appendix E. Study Tables**

**Table 1. Sources where respondents had heard of SafeScript NSW**

| Sources* (n=24) | n (%) |
| --- | --- |
| Medical practice/specialist clinic I attend | 9 (37.5) |
| Other (Optional free-text) | 7 (29.2) |
| Internet | 5 (20.8) |
| Word of mouth | 5 (20.8) |
| Newsletter from consumer bodies/groups | 3 (12.5) |

Note. * = multiple selections allowed

**Table 2. Knowledge of SafeScript NSW**

| Rating (n=23) | n (%) |
| --- | --- |
| None | 7 (30.4) |
| Fair | 7 (30.4) |
| Good | 3 (13) |
| Very Good | 3 (13) |
| Excellent | 3 (13) |

**Table 3. Discussions with prescriber or pharmacist regarding findings from SafeScript NSW**

| Discussion (Total n=22) | n (%) |
| --- | --- |
| Yes | 4 (18.2) |
| No | 13 (59.1) |
| Not sure | 5 (22.7) |
